# Effect of supervised exercise training during pregnancy on neonatal and maternal outcomes among overweight and obese women. Secondary analyses of the ETIP trial: A randomised controlled trial

**DOI:** 10.1371/journal.pone.0173937

**Published:** 2017-03-21

**Authors:** Kirsti Krohn Garnæs, Siri Ann Nyrnes, Kjell Åsmund Salvesen, Øyvind Salvesen, Siv Mørkved, Trine Moholdt

**Affiliations:** 1 Department of Circulation and Medical Imaging, NTNU, Norwegian University of Science and Technology, Trondheim, Norway; 2 Department of Paediatrics, St. Olavs Hospital, Trondheim University Hospital, Trondheim, Norway; 3 Department of Obstetrics and Gynaecology, St. Olavs Hospital, Trondheim University Hospital, Trondheim, Norway; 4 Department of Laboratory Medicine, Children's and Women's Health, Norwegian University of Science and Technology, Trondheim, Norway; 5 Department of Public Health and General Practice, NTNU, Norwegian University of Science and Technology, Trondheim, Norway; 6 Clinical Service, St. Olavs Hospital, Trondheim University Hospital, Trondheim, Norway; University of Tennessee Health Science Center, UNITED STATES

## Abstract

**Background:**

Maternal obesity associates with complications during pregnancy and childbirth. Our aim was to investigate if exercise during pregnancy in overweight/obese women could influence birth weight or other neonatal and maternal outcomes at delivery.

**Material and methods:**

This is a secondary analysis of a randomised controlled trial of exercise training in pregnancy for women with body mass index (BMI) ≥ 28 kg/m^2^. Ninety-one women (31.3 ± 4.3 years, BMI 34.5 ± 4.2 kg/m^2^) were allocated 1:1 to supervised exercise during pregnancy or to standard care. The exercise group was offered three weekly training sessions consisting of 35 minutes of moderate intensity walking/running followed by 25 minutes of strength training. Data from 74 women (exercise 38, control 36) were analysed at delivery.

**Results:**

Birth weight was 3719 ± 695 g in the exercise group and 3912 ± 413 g in the control group (CI -460.96, 74.89, *p* = 0.16). Birth weight > 4000 g was 35% in the exercise group and 52% in the control group (*p* = 0.16). Mean gestational age at delivery was 39.1 weeks in the exercise group and 39.5 weeks in the control group (CI -1.33, 0.43, *p* = 0.31). No significant between-group differences were found in neonatal body size, skinfold thickness, placental weight ratio, or Apgar score. The prevalence of caesarean section was 24% in the exercise group and 17% in the control group (CI 0.20, 2.05, *p* = 0.57). Mean length of hospital stay was 4.8 days in the exercise group and 4.5 days in the control group (CI -0.45, 1.00, *p* = 0.45).

**Conclusions:**

Offering supervised exercise during pregnancy for overweight and obese women did not influence birth weight or other neonatal and maternal outcomes at delivery. However our trial was limited by low sample size and poor adherence to the exercise protocol, and further research is needed.

**Trial registration:**

ClinicalTrials.gov NCT01243554

## Introduction

The prevalence of maternal overweight and obesity is increasing [1] and has important consequences for the health of mother and child at delivery [2]. The World Health Organization (WHO) classifies overweight as body mass index (BMI) ≥ 25 kg/m^2^, and obesity as BMI ≥ 30 kg/m^2^ [3]. Overweight and obesity in pregnancy is associated with adverse neonatal outcomes as high birth weight [4], preterm birth, perinatal death, congenital anomalies, birth trauma related to macrosomia, and transfer to neonatal intensive care unit (NICU) [5, 6]. Adverse outcomes for the mother at delivery include need for caesarean section and prolonged hospital stay [5, 7–9]. Furthermore, overweight and obese pregnant women are at increased risk for reduced insulin sensitivity and subsequently high levels of circulating glucose in the foetus [10–13]. This can lead to foetal overgrowth and macrosomia (birth weight ≥ 4000 g) and is associated with adverse obstetrical outcomes [14] childhood obesity, and cardiometabolic diseases later in life [10–13, 15, 16]. Long-term consequences of these adverse outcomes have led to an increased attention towards maternal obesity as a contributing factor to the developmental origins of health and disease [11, 17].

Previous studies have reported positive effects of lifestyle interventions combining diet and exercise on some delivery-related outcomes in normal weight women, including reduced risks of preeclampsia, shoulder dystocia and preterm birth [18]. However, the effect of lifestyle interventions in pregnancy on birth weight, gestational age, rates of caesarean section, and transfer to NICU are still unclear [18–21]. Wiebe et al. [22] found in a meta-analysis that supervised prenatal exercise reduced neonatal birth weight and caesarean delivery among women with BMI ≤ 24.9. Previous studies have reported limited effect of lifestyle interventions on maternal or neonatal outcomes at delivery in overweight and obese women [19, 23–25].

We have previously published data from a randomised controlled trial of exercise training in pregnancy for women with BMI ≥ 28 kg/m^2^, addressing effects of regular exercise on gestational weight gain and several secondary outcomes in late pregnancy [26]. In the present paper we report secondary analyses of maternal and neonatal outcomes at delivery. We aimed to investigate the effect of regular exercise during pregnancy on birth weight, and hypothesised that the birth weight in the exercise group would be lower compared to the control group. In addition, we investigated possible effects of exercise training on neonatal outcomes such as body composition, Apgar score, placental weight ratio, preterm birth and admission to neonatal intensive care unit, and maternal outcomes such as mode of delivery, perineal tears and length of hospital stay.

## Materials and methods

### Trial design

The Exercise Training in Pregnancy (ETIP) trial was a single centre, parallel group randomised controlled trial. The trial included women with pre-pregnancy BMI ≥ 28 kg/m^2^. The trial was carried out at The Norwegian University of Science and Technology, NTNU, and St. Olavs Hospital, University Hospital in Trondheim, Norway, with participant recruitment from September 2010 to March 2015. The trial was approved by the Regional Committee for Medical and Health Research Ethics (REK midt 2010/1522), and registered in ClinicalTrials.gov (NCT01243554). The protocol and the results from primary outcomes in the trial have been published previously [26, 27].

We made changes to the study protocol after commencement of the trial [26]. In November 2012 the criterion for maximum inclusion time gestational week 16 was changed to gestational week 18, and in March 2013 the inclusion criterion pre-pregnancy BMI ≥ 30 kg/m^2^ was changed to BMI ≥ 28 kg/m^2^. The changes were done to accommodate slow recruitment in the trial, and the revised study protocol was approved by the Regional Committee for Medical and Health Research Ethics.

### Participants

Inclusion criteria were pre-pregnancy BMI ≥ 28 kg/m^2^, age ≥ 18 years, carrying a singleton viable foetus at 11–14 gestational weeks. Also, the women had to be able to visit St. Olavs Hospital for assessments and exercise sessions. Data on pre-pregnancy BMI was based on self-reported weight and height. Exclusion criteria were diseases affecting participation [28], high risk for preterm delivery [28], and regularly exercise training (twice or more weekly) in the period before inclusion. The procedures were in accordance to ethical standards of research and the Helsinki Declaration. Women were recruited through Google advertisements and by notices enclosed with invitations for routine ultrasound scans at St. Olavs Hospital. At the time of recruitment and before randomisation and participation, the women received written information and signed an informed consent.

### Intervention

All participants received standard maternity care, and women in the control group were not discouraged from physical activity. Women in the exercise group were offered supervised exercise sessions at St. Olavs Hospital three times weekly from early pregnancy until delivery. The exercise program was in accordance with the recommendations from the American College of Obstetricians and Gynaecologists [29]. The exercise sessions were supervised by a physical therapist and lasted for 60 min, and consisted of 35 min of walking/jogging on a treadmill for (endurance training), and 25 min of resistance training for large muscle groups and the pelvic floor muscles [26, 27]. The intensity of the endurance training was moderate, set to ~80% of maximal capacity (corresponding to Borg scale 12–15) [30]. The resistance training was with use of own body weight and consisted of squats, diagonal lifts on all fours, push-ups, oblique abdominal crunches and the “plank exercise”. Each exercise was performed as three sets of ten repetitions separated by a 1-min rest between sets; the “plank exercise” was performed in 30 sec. The pelvic floor exercises consisted of three sets of ten repetitions of pulling the pelvic floor up and holding the contraction for 6–8 s. The exercise program and load were individually adjusted when needed.

In addition to the supervised program women were asked to do a 50 minutes home exercise program twice weekly and to do pelvic floor muscle exercises every day. Adherence to the exercise program was registered in a training diary. The participants in the exercise group received a weight gain curve of recommended weight gain during pregnancy according to the 2009 Institute of Medicine recommendations [31]. The intervention group received no dietary advice, but both groups received a standard brochure at inclusion with information about healthy living during pregnancy.

### Outcomes

Baseline assessments were undertaken in gestational week 12–18 (early pregnancy). Neonatal and maternal outcomes in the current article were assessed at delivery and during the maternity stay at the hospital.

Birth weight was the principal outcome in this secondary analysis. Other neonatal outcomes were birth weight > 4000 g, head circumference, length, body surface area (BSA), skinfold thickness, abdominal and upper arm circumference, gestational age, Apgar score at 1 and 5 minutes, placental weight and placental weight ratio (PWR), and transfer to Neonatal Intensive Care Unit (NICU). Maternal outcomes were mode of delivery, preterm birth, preeclampsia, perineal tears, and duration of hospital stay.

Birth weight was measured at delivery by a Seca baby weight (Medema, Norway) by the birth attendants. We measured skinfold thickness by a Harpenden Skinfold Calliper (Holtain, Ltd, UK), on the right side of the body at the following sites; subscapularis; at the bottom of the angelus inferior scapula, triceps; at the middle between the olecranon and the humeral head. We used a measuring tape to measure abdominal and upper arm circumference. The abdomen was measured at the level of umbilicus and the upper arm at the middle between olecranon and humeral head. Skinfold thickness and circumference measurement were undertaken by the first author (KKG).

Mode of delivery, perineal tears, hospital stay, new-born length, head circumference, gestational age, Apgar score, placenta weight, and transfer to NICU were recorded in the hospital records. We calculated BMI of the newborn as weight in kilograms divided by the square of height in meters, and Body Surface Area (BSA, in m^2^) by the Mosteller Formula as (height (cm) x weight (kg) /3600)^1/2^ [32]. We calculated the ratio of birth weight and placental weight (placental weight ratio, PWR) as placenta weight divided by birth weight. We defined preterm birth as delivery before gestational week 37.

### Sample size

We calculated the sample size in the ETIP trial based on the primary outcome; gestational weight gain from baseline to delivery [26]. We assumed a 6 kg mean difference between groups as clinical relevant [33, 34]. A two-sided independent sample *t*-test with a 5% level of significance, a standard deviation of 10, and a power of 0.9 gave a target study population of 59 in each group. We estimated the dropout to be 15% and aimed to include 150 women. We did not do an *a priori* sample size calculation for the outcomes reported in this paper, but we did a post-hoc power calculation on birth weight. Based on previous trials [35, 36], we considered a mean difference in birth weight 250 g clinically relevant, based on previous studies. With standard deviation of 430 g, alpha 0.05 and beta 0.2, we would have needed 94 participants in the trial to demonstrate a difference in birth weight between groups.

### Randomisation and allocation

Trial participants were randomised 1:1 to exercise or control groups after baseline assessments. We used a computer random number generator developed and administrated at the Unit for Applied Clinical Research at NTNU to generate the random allocation sequence, as previously detailed [26].

### Blinding

Birth attendants were blinded for group allocation. Measurements of skinfold thickness, abdominal circumference, and intervention administration were done non-blinded by the investigators. The first author (KKG) was not blinded for group allocation as she supervised the exercise training. The statistician was blinded for group allocation.

### Statistical methods

The principal analyses were based on the intention to treat principle and all available data were used at all time points. Continuous data were tested for normality, and we used independent samples t-tests to assess the effect of the intervention. We used the Fisher’s Exact Test or Pearson Chi Square Test to analyse effects of the intervention on dichotomous outcomes, with the exercise group as the reference group. Due to the randomisation model, we assumed no systematic differences between groups at baseline, however differences between groups at baseline were tested [26].

We performed supplementary analyses where we adjusted for gestational age and parity. We also analysed the association between BMI at early pregnancy and the variables birth weight and risk for caesarean delivery.

In addition to the primary analyses, we performed per protocol analyses where we compared women in the exercise group adhering to the exercise protocol with the control group [27]. Exercise per protocol was defined as one of the following: 1) attending ≥ 42 organized exercise sessions, 2) attending ≥ 28 exercise sessions + performing ≥ 28 home exercise sessions, or 3) performing ≥ 60 home exercise sessions. To count as a home session, the exercise had to be ≥ 50 minutes of either aerobic and/or strength training.

For the statistical analyses, we used IBM SPSS Statistics 23 for outcomes at delivery. Baseline demographic characteristics were analysed by Stata version 13.1. Supplementary analysis including adjustments and associations were analysed by R version 2.13.1. In comparison between groups we report mean values with 95% confidence intervals, for the continuous variables and odds ratios with 95% confidence intervals for dichotomous variables. We considered p-values < 0.05 as significant.

## Results

**Fig 1:**
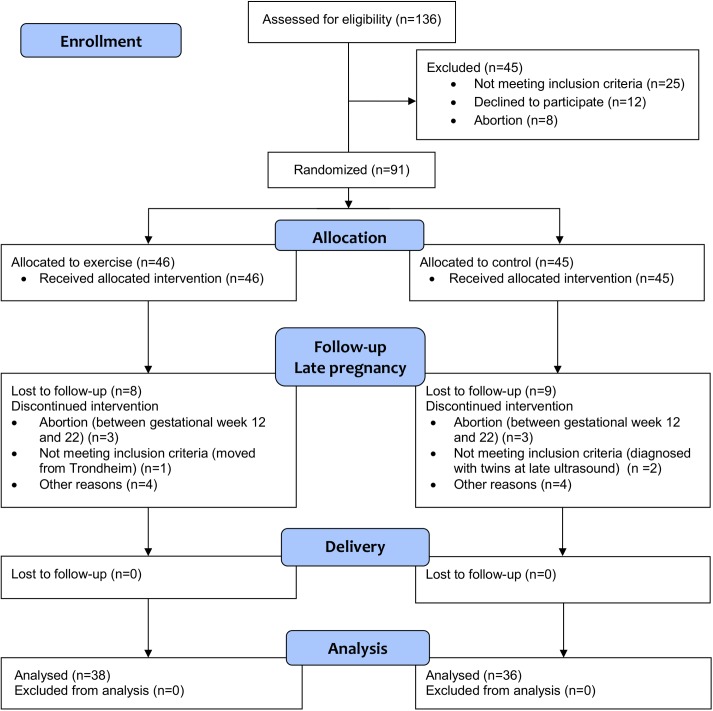
Flow chart of the ETIP trial (CONSORT flow diagram).

Fig 1 shows the participant flow in the ETIP trial. Complete baseline data has been previously published [26]. No differences between groups at baseline were found, except from significant lower fasting glucose in the exercise group, 4.6 mmol/l vs 5.0 mmol/l, *p* = 0.02 [26]. Mean pre-pregnancy (self-reported) BMI was 33.9 ± 3.8 kg/m^2^ in the exercise group, and 35.1 ± 4.6 kg/m^2^ in the control group. Mean gestational weight gain was 10.5 kg in the exercise group, and 9.2 kg in the control group (*p* = 0.35). Fifty-eight percent of the women in the exercise group, and 44% of the women in the control group, gained more weight than recommended by the IOM guidelines.

Seventy-four women (exercise 38, control 36) were included in the analyses at delivery. Two women in the exercise group (BMI 29.7 and 28.3 kg/m^2^), and three women in the control group (BMI 29.4, 28.8 and 29.7 kg/m^2^), were classified as overweight at baseline. All other women had a pre-pregnancy BMI ≥ 30 kg/m^2^, and were classified as obese. Fifty percent of women in the exercise group and 38% in the control group were nulliparous (*p* = 0.18 Women in the intervention group performed 31.7 ± 15.3 (range 0–53) supervised sessions at the hospital and 19.2 ± 16.5 (range 0–72) exercise sessions at home.

### Neonatal outcomes

We found no significant differences in birth weight or other neonatal outcomes at delivery between the exercise group and the control group (Table 1).

**Table 1:** Neonatal outcomes at delivery for the exercise- and the control group. Continuous data is presented as mean and standard deviation (SD) with comparison between groups as mean difference with 95% confidence interval (CI) and p-value. Dichotomous data is presented as number (n) and percent (%) and comparison between groups as odds ratio (OR), with 95% CI and p-value.

Neonatal outcomes	Exercise groupN = 38		Control groupN = 36		Between-group differences		
	*n*	*Mean* ± *SD*	*n*	*Mean* ± *SD*	*Mean diff*	*95% CI*	*p-value*^*a*^
Birth weight (g)	37	3719 ± 695	36	3912 ± 413	-193.04	-460.96, 74.89	0.16
Gestational age (weeks)^b^	37	39.1 ± 2.3	36	39.5 ± 1.3	-0.45	-1.33, 0.43	0.31
Length (cm)	34	50.7 ± 1.7	36	51.1 ± 1.9	-0.46	-1.32, 0.39	0.28
Head circumference (cm)	35	35.9 ± 1.5	36	35.8 ± 1.5	0.18	-0.53, 0.90	0.61
Abdominal circumference (cm)	32	32.1 ± 2.5	30	31.9 ± 2.1	0.12	-1.03, 1.28	0.83
Upper arm circumference (cm)	32	11.4 ± 2.1	31	11.4 ± 1.0	0.03	-0.79, 0.84	0.95
Body mass index at birth (kg/m^2^)	34	14.9 ± 1.3	36	15.0 ± 1.3	-0.06	-0.67, 0.56	0.86
Body surface area (m^2^)	34	0.23 ± 0.02	36	0.24 ± 0.02	-0.003	-0.011, 0.004	0.38
Skinfold thickness triceps	32	6.1 ± 2.0	30	6.3 ± 2.1	-0.25	-1.30, 0.79	0.63
Skinfold thickness subscapularis	32	5.4 ± 1.5	29	5.7 ± 1.9	-0.26	-1.13, 0.60	0.55
Apgar score 1 minute	36	8.4 ± 1.1	34	8.3 ± 1.7	0.15	-0.53, 0.83	0.66
Apgar score 5 minute	36	9.6 ± 0.5	34	9.4 ± 1.2	0.20	-0.24, 0.64	0.37
Placenta weight (g)	34	705.8 ± 165.4	32	666.7 ± 128.6	39.10	-34.07, 112.28	0.29
Placental weight ratio^c^	34	0.18 ± 0.02	32	0.17 ± 0.03	0.01	-0.001, 0.023	0.08
		*n (%)*		*n (%)*	*OR*	*95% CI*	*p-value*^*a*^
Birth weight > 4000 g	37	13 (35)	36	19 (53)	1.4	0.88, 2.36	0.16
Transfer to NICU^d^	37	3 (8)	34	3 (9)	1.0	0.46, 2.41	0.91
Preterm birth^e^	38	2 (5)	36	0 (0)	-	-	0.49

^a^Continuous variables were analysed by Independent Samples t-test. Dichotomous variables were analysed by Fisher’s Exact Test and Pearson Chi-Square. Apgar score was analysed by Nonparametric Tests, Mann-Whitney U.

^b^Weeks between the first day of the mother’s last menstrual period and the day of delivery.

^c^Placenta weight divided by birth weight.

^d^Neonatal Intensive Care Unit

^e^Delivery before gestational week 37.

There were two cases of preterm birth (pregnancy weeks 29 and 34) in the exercise group (Table 1), which represented two cases of neonatal birth weight < 2500 g. These women had their last exercise sessions 9 and 14 days prior to the preterm births. One woman in the exercise group chose to terminate the pregnancy at week 19+5 due to severe foetal malformations diagnosed at a routine second trimester ultrasound scan.

Three neonates in each group needed admission to the NICU after birth. In the exercise group, two neonates were admitted due to prematurity and one due to meconium aspiration. In the control group one neonate was transferred to NICU due to hypoglycaemia and infection, one due to asphyxia caused by shoulder dystocia, complicated by a humerus fracture, and one due to persistent pulmonary hypertension. Two women, one in each group, delivered their babies at other hospitals than St. Olavs Hospital, and we therefore have missing data on their outcomes.

### Maternal outcomes

No significant differences between groups were seen in length of hospital stay, mode of delivery, or perineal tears. Approximately 2/3 of the women in both groups had a normal delivery (Table 2). No women in the exercise group and two women in the control group had preeclampsia (*p* = 0.24).

**Table 2:** Maternal outcomes at delivery for the exercise- and the control group. Continuous data is presented as mean and standard deviation (SD), with comparison between groups are as mean difference with 95% confidence interval (CI) and p-value. Dichotomous data is presented as number (*n*) and percent (%), with comparison between groups as odds ratio (OR), with 95% CI and p-value.

Maternal Outcomes	Exercise groupn = 38	Control groupn = 36	Between-group differences		
	*n (%)*	*n (%)*	*OR*	*95% CI*	*p-value*^a^
Mode of delivery^b^					
Normal vaginal delivery	22 (60)	24 (69)	1.2	0.77, 1.89	0.47
Operative vaginal delivery	7 (19)	5 (14)	0.9	0.50, 1.47	0.75
Caesarean section	9 (24)	6 (17)	0.8	0.50, 1.33	0.57
Perineal tears, grade 3–4^c^	4 (18)	2 (10)	0.7	0.08, 2.91	0.66
	*Mean* ± *SD*	*Mean* ± *SD*	*Mean diff*	*95% CI*	*p-value*^*a*^
Length of hospital stay (days)^d^	4.8 ± 1.6	4.5 ± 1.5	0.28	-0.45, 1.00	0.45

^a^Continuous variables were analysed by Independent Samples t-test, dichotomous variables by Fisher’s Exact Test and Pearson Chi-Square.

^b^One missing in each group.

^c^Out of women with normal vaginal delivery. Three missing in the control group.

^d^Four missing in the exercise group and five in the control group.

### Additional analyses

We did secondary analyses controlling for parity and gestational age and found no statistically significant difference between groups. We also analysed possible associations between BMI at early pregnancy with birth weight and risk for caesarean delivery and found no statistically significant associations.

In secondary per protocol analyses we compared the control group to the women in the exercise group who adhered to the exercise protocol (n = 19, 50%). No significant differences in birth weight between exercise (3742 g ± 652) and control groups (3912 g ± 413), *p* = 0.24 were observed (S1 Table). There were no significant differences between groups in any other maternal and neonatal outcomes in the per protocol analysis (S1 and S2 Tables).

## Discussion

### Main findings

We found no effect of regular supervised exercise training during pregnancy on birth weight, body composition, or size of the neonate. Furthermore, we observed no between-group differences in any other maternal or neonatal outcomes at delivery.

### Neonatal outcomes

We found no difference between groups in neonatal birth weight, body surface area or body composition. This is in line with several other studies [18, 21, 37, 38] and confirmed by two recent systematic reviews [23, 24]. However, Barakat and colleagues [39] found a 2.5 times higher risk of macrosomia in women allocated to a control group compared to women who adhered to ≥ 80% to a supervised exercise program of three weekly sessions during pregnancy. In line with this, Hopkins et al. [36] observed significantly lower birth weight among babies born to women who exercised during pregnancy. Both these studies included women of all BMI categories. We observed a tendency of higher prevalence of children with birth weight > 4000 g in the control group, but the difference was not statistically significant. This finding is supported by a meta-analysis of 5278 newborns born to women in all BMI categories, in which a lower prevalence of macrosomia, despite no difference in birth weight, was observed in women who followed a lifestyle intervention program in pregnancy [40]. Of note, about 50% of the children born to women in the control group had birth weight > 4000 g. Babies with birth weight ≥ 4000 g are at increased risk for birth complications, childhood obesity, adult obesity, and future metabolic syndrome, compared to babies with birth weight 2500–4000 g [41].

Comparing the intervention protocol in the ETIP trial to exercise interventions in other randomised trials shows that many studies, as the ETIP trial, base their protocol on the American College of Obstetricians and Gynaecologists [29] recommendations for physical activity during pregnancy. Data from overweight and obese women in comparable RCTs of Barakat et al. [42] and Nascimento et al. [43], showed no effect on neonatal birth weight. Exercise interventions showing most effect on neonatal birth weight are characterized by including all weight classes [21, 42, 44, 45], and by including the participants early in pregnancy (gestational week 6–13) [42, 45], or by high frequency of exercise sessions (five times per week) [44]. Most RCTs on exercise training in pregnancy make use a combination of endurance training at light to moderate intensity, and resistance training, with duration of the sessions of 45–60 minutes two-three times per week until gestational week 36–38. The amount, intensity and duration of exercise in the ETIP trial are in line with several other randomised controlled trials on exercise in pregnancy, however, the mean inclusion time (gestational week) was higher (16.4) in the ETIP trial, and thus decreases the number of weeks of exercise during the pregnancy.

We observed a borderline statistically significant difference in PWR between groups (*p* = 0.08), with means in both groups within the normal range of PWR [46]. We prefer not to speculate too much around a non-significant result, but placental weight is a measure that can reflect several aspects of foetal growth. Furthermore, both low and high PWR can predict adverse neonatal outcomes at delivery [47], and high PWR has been found to associate with increased risk of obesity and cardiovascular disease later in life [48–50].

We found no differences between groups in neonatal outcomes at delivery. This is in line with several other studies reporting no effects of exercise- or lifestyle (combining diet and exercise) interventions during pregnancy on Apgar score or head circumference [21, 37, 51]. Haakstad & Bø [21] observed higher mean Apgar scores at 1 minute, but not at 5 minutes, among newborns born to women allocated to training in a randomised controlled trial of 105 women. This was observed in a per protocol analysis and not in the intention-to treat analysis, and Apgar score at 5 minutes is considered a better sign of newborn wellbeing than Apgar score at 1 minute [52, 53].

### Maternal outcomes

Most women had a normal vaginal delivery, and we observed no significant effect of exercise training on mode of delivery. A meta-analysis of 10 trials with a total of 3160 women found more normal deliveries among healthy regularly exercising pregnant women in all BMI categories [19]. However, a meta-analysis and systematic review of 6 RCTs (n = 2762) of combined diet and exercise intervention during pregnancy among overweight and obese women found no effect on mode of delivery [23].

### Strengths

The exercise intervention in our trial included supervised training sessions. Supervised training sessions are important for compliance and effect of the intervention [54]. We used exercise as the only intervention, thereby enabling us to assess the isolated effects of exercise training on the reported outcomes. We included previously sedentary women with a BMI of 28 or more in the trial, hence our study population can be considered homogeneous. All data regarding delivery information was collected from patients’ records at St. Olavs Hospital, and the records were assessed by personnel blinded for allocation. We also regard the additional measurements of skinfold thickness, body surface area and abdominal circumference as strengths to our study.

### Limitations

The main limitation of our trial is the small number of participants and hence the risk for statistical type 2 error. We planned to include 150 women in the ETIP trial [27], however ended up with 91 randomised women after a prolonged inclusion time. Furthermore, since only 50% of the women adhered to the training protocol, potential effects of the intervention may be undetected. However, the adherence to protocol was similar to other comparable trials on effects of lifestyle changes in pregnancy. When interpreting the per protocol analysis, care must be taken due to the risk of selection bias as compliance with the exercise program could be associated with other prognostic factors.

To accommodate slow recruitment to the trial, we prolonged the time limit for inclusion in the trial with two weeks (until pregnancy week 18). This reduced the time for training adaptations to occur, and may have reduced the chance for detecting effects of the intervention. In addition, we changed pre-pregnancy BMI limit from ≥ 30.0 to ≥ 28.0 kg/m^2^, and thereby including five overweight women in the analysis at delivery. This change to the protocol may have affected the homogeneity of the trial population, reduced the mean BMI in both groups, and thereby somewhat reduced the risk for adverse events. We argue, however, that including five women with a BMI between 28.0 and 30.0 kg/m^2^ will not be of major importance for the interpretation of our results.

Further, the control group attended quite comprehensive health assessments during the pregnancy, and therefore may have increased their awareness of healthy living during the pregnancy.

We acknowledge that not providing any information regarding the participants’ diet during the pregnancy is a limitation. Maternal nutrition may be a confounding factor due to its effect on both neonatal and maternal outcomes. We can assume that the women in the exercise group would be extra motivated for eating healthy as part of a more healthy lifestyle. On the other side, they could also compensate for the energy expended through exercise by eating more [55].

Measurement of neonatal skinfold thickness was non-blinded and may have introduced bias to the data on the effect of exercise on body composition. Interpretation must be done with caution.

### Generalisability

The ETIP trial had few exclusion criteria, and offered training sessions at different times of the day, indicating that a large proportion of pregnant women could volunteer for participation. We included about 10% of eligible women with BMI ≥ 28 in the area of St. Olavs Hospital, which is a similar inclusion rate as a previous RCT (TRIP trial) on exercise in pregnancy conducted in the same area a few years earlier [56]. These women were found to be representative for the population of pregnant women, and it is likely that this responds to the ETIP trial too. However, it is possible that the women recruited to the ETIP trial were extra aware of the possible benefits of lifestyle changes on maternal and neonatal health, and therefore more motivated to exercise during pregnancy compared to women who did not volunteer for this trial.

Comparison between the results from the current trial and data from large cohort studies [57–59] on women with pre-pregnancy obesity shows that the number of adverse outcomes in our control group was low and indicates that we had a quite healthy study population in the ETIP trial.

### Clinical relevance

The number of obese pregnant women is increasing. Thus, we urgently need to establish strategies to prevent associated risk factors. The intervention used in the ETIP trial was based on recommendations for physical activity during pregnancy and involved an exercise program that can easily be performed individually or in groups, at home or supervised, without any equipment.

There were no adverse events in our trial related to the exercise intervention. Some previous studies have reported high risk of preterm delivery associated with exercise during pregnancy [60], but a recent meta-analysis did not find any association between aerobic exercise for 35–90 minutes 3–4 times per week and increased risk of preterm birth [61]. In the current trial two women in the exercise group had a preterm delivery, and we found no indication of this being related to participating in the exercise program. The majority of the women in this trial had a normal delivery, and the rate of caesarean delivery was relatively low.

## Conclusions

We found no effect of offering regularly supervised exercise training during pregnancy on birth weight or body size of neonates born to women with a pre-pregnancy body mass index of 28 kg/m^2^ or more. The intervention program had no impact on other neonatal and maternal outcomes at delivery. Our trial was limited by small sample size and low adherence to the exercise protocol. We need larger, well-designed RCTs to further investigate the effect of exercise training on neonatal and maternal outcomes in this population.

## Supporting information

S1 Table Supplementary Table 1. Neonatal outcomes at delivery for the per-protocol exercise group and the control group. Continuous data is presented as mean and standard deviation (SD) with comparison between groups as mean difference with 95% confidence interval (CI) and p-value. Dichotomous data is presented as number (n) and percent (%) and comparison between groups as odds ratio (OR), with 95% confidence interval (CI) and p-value.
(DOCX)

S2 Table Supplementary Table 2. Maternal outcomes at delivery for the per-protocol exercise group and the control group. Continuous data is presented as mean and standard deviation (SD), with comparison between groups are as mean difference with 95% confidence interval (CI) and p-value. Dichotomous data is presented as number (*n*) and percent (%), with comparison between groups as odds ratio (OR), with 95% confidence interval (CI) and p-value.
(DOCX)

S3 Table Supplementary Table 3. Baseline characteristics of all women included in the ETIP study.
(DOCX)

## References

[1] American Diabetes A. Diagnosis and classification of diabetes mellitus. Diabetes Care. 2010;33 Suppl 1:S62–9. PubMed Central PMCID: PMC2797383. 20042775 10.2337/dc10-S062 PMC2797383

[2] Catalano PM, McIntyre HD, Cruickshank JK, McCance DR, Dyer AR, Metzger BE, et al The hyperglycemia and adverse pregnancy outcome study: associations of GDM and obesity with pregnancy outcomes. Diabetes Care. 2012;35(4):780–6. Epub 2012/02/24. PubMed Central PMCID: PMCPMC3308300. 10.2337/dc11-1790 22357187 PMC3308300

[3] World Health Organization W. Obesity and overweight. http://www.who.int/mediacentre/factsheets/fs311/en/: 2016 June 2016.

[4] Gaudet L, Ferraro ZM, Wen SW, Walker M. Maternal obesity and occurrence of fetal macrosomia: a systematic review and meta-analysis. BioMed research international. 2014;2014:640291 Epub 2014/12/30. PubMed Central PMCID: PMCPMC4273542. 10.1155/2014/640291 25544943 PMC4273542

[5] Kim SS, Zhu Y, Grantz KL, Hinkle SN, Chen Z, Wallace ME, et al Obstetric and Neonatal Risks Among Obese Women Without Chronic Disease. Obstet Gynecol. 2016;128(1):104–12. Epub 2016/06/09. PubMed Central PMCID: PMCPMC4917420. 10.1097/AOG.0000000000001465 27275800 PMC4917420

[6] Cnattingius S, Bergstrom R, Lipworth L, Kramer MS. Prepregnancy weight and the risk of adverse pregnancy outcomes. The New England journal of medicine. 1998;338(3):147–52. Epub 1998/01/15. 10.1056/NEJM199801153380302 9428815

[7] Lutsiv O, Math J, Beyene J, McDonald SD. The effects of morbid obesity on maternal and neonatal health outcomes: a systematic review and meta-analyses. Obes Rev. 2015;7;16(7):531–46:531–46. Epub 2015 Apr 24. 10.1111/obr.12283 25912896

[8] Oken E, Kleinman KP, Belfort MB, Hammitt JK, Gillman MW. Associations of gestational weight gain with short- and longer-term maternal and child health outcomes. Am J Epidemiol. 2009;170(2):173–80. Epub 2009/05/15. PubMed Central PMCID: PMC2727269. 10.1093/aje/kwp101 19439579 PMC2727269

[9] Weiss JL, Malone FD, Emig D, Ball RH, Nyberg DA, Comstock CH, et al Obesity, obstetric complications and cesarean delivery rate—a population-based screening study. Am J Obstet Gynecol. 2004;190(4):1091–7. Epub 2004/05/01. 10.1016/j.ajog.2003.09.058 15118648

[10] Barker DJ. The developmental origins of adult disease. J Am Coll Nutr. 2004;23(6 Suppl):588S–95S. Epub 2005/01/11. 15640511 10.1080/07315724.2004.10719428

[11] Waterland RA, Garza C. Potential mechanisms of metabolic imprinting that lead to chronic disease. Am J Clin Nutr. 1999;69(2):179–97. 9989679 10.1093/ajcn/69.2.179

[12] Frias AE, Grove KL. Obesity: a transgenerational problem linked to nutrition during pregnancy. Semin Reprod Med. 2012;30(6):472–8. PubMed Central PMCID: PMC3615704. 10.1055/s-0032-1328875 23074005 PMC3615704

[13] Tan HC, Roberts J, Catov J, Krishnamurthy R, Shypailo R, Bacha F. Mother's pre-pregnancy BMI is an important determinant of adverse cardiometabolic risk in childhood. Pediatr Diabetes. 2015;16(6):419–26. Epub 2015/03/25. PubMed Central PMCID: PMCPMC4534350. 10.1111/pedi.12273 25800542 PMC4534350

[14] Boulet SL, Salihu HM, Alexander GR. Mode of delivery and birth outcomes of macrosomic infants. J Obstet Gynaecol. 2004;24(6):622–9. 10.1080/01443610400007828 16147599

[15] Nehring I, Schmoll S, Beyerlein A, Hauner H, von Kries R. Gestational weight gain and long-term postpartum weight retention: a meta-analysis. The American journal of clinical nutrition. 2011;94(5):1225–31. Epub 2011/09/16. 10.3945/ajcn.111.015289 21918221

[16] Siega-Riz AM, Viswanathan M, Moos MK, Deierlein A, Mumford S, Knaack J, et al A systematic review of outcomes of maternal weight gain according to the Institute of Medicine recommendations: birthweight, fetal growth, and postpartum weight retention. Am J Obstet Gynecol. 2009;201(4):339 e1–14. 19788965 10.1016/j.ajog.2009.07.002

[17] Drake AJ, Reynolds RM. Impact of maternal obesity on offspring obesity and cardiometabolic disease risk. Reproduction. 2010;140(3):387–98. Epub 2010/06/22. 10.1530/REP-10-0077 20562299

[18] Thangaratinam S, Rogozinska E, Jolly K, Glinkowski S, Roseboom T, Tomlinson JW, et al Effects of interventions in pregnancy on maternal weight and obstetric outcomes: meta-analysis of randomised evidence. BMJ. 2012;344:e2088 Epub 2012/05/19. PubMed Central PMCID: PMCPMC3355191. 10.1136/bmj.e2088 22596383 PMC3355191

[19] Poyatos-Leon R, Garcia-Hermoso A, Sanabria-Martinez G, Alvarez-Bueno C, Sanchez-Lopez M, Martinez-Vizcaino V. Effects of exercise during pregnancy on mode of delivery: a meta-analysis. Acta Obstet Gynecol Scand. 2015;94(10):1039–47. Epub 2015/05/13. 10.1111/aogs.12675 25965378

[20] Perales M, Santos-Lozano A, Ruiz JR, Lucia A, Barakat R. Benefits of aerobic or resistance training during pregnancy on maternal health and perinatal outcomes: A systematic review. Early Hum Dev. 2016;94:43–8. 10.1016/j.earlhumdev.2016.01.004 26850782

[21] Haakstad LA, Bo K. Exercise in pregnant women and birth weight: a randomized controlled trial. BMC Pregnancy Childbirth. 2011;11:66 Epub 2011/10/04. PubMed Central PMCID: PMCPMC3198740. 10.1186/1471-2393-11-66 21961534 PMC3198740

[22] Wiebe HW, Boule NG, Chari R, Davenport MH. The effect of supervised prenatal exercise on fetal growth: a meta-analysis. Obstet Gynecol. 2015;125(5):1185–94. Epub 2015/05/02. 10.1097/AOG.0000000000000801 25932847

[23] Oteng-Ntim E, Varma R, Croker H, Poston L, Doyle P. Lifestyle interventions for overweight and obese pregnant women to improve pregnancy outcome: systematic review and meta-analysis. BMC Med. 2012;10:47 PubMed Central PMCID: PMC3355057. 10.1186/1741-7015-10-47 22574949 PMC3355057

[24] Sui Z, Grivell RM, Dodd JM. Antenatal exercise to improve outcomes in overweight or obese women: A systematic review. Acta Obstet Gynecol Scand. 2012;91(5):538–45. Epub 2012/01/11. 10.1111/j.1600-0412.2012.01357.x 22229625

[25] Barakat R, Pelaez M, Cordero Y, Perales M, Lopez C, Coteron J, et al Exercise during pregnancy protects against hypertension and macrosomia: randomized clinical trial. Am J Obstet Gynecol. 2015. 10.1016/j.ajog.2015.11.039 26704894

[26] Garnaes KK, Morkved S, Salvesen O, Moholdt T. Exercise Training and Weight Gain in Obese Pregnant Women: A Randomized Controlled Trial (ETIP Trial). PLoS Med. 2016;13(7):e1002079 PubMed Central PMCID: PMCPMC4961392. 10.1371/journal.pmed.1002079 27459375 PMC4961392

[27] Moholdt TT, Salvesen K, Ingul CB, Vik T, Oken E, Morkved S. Exercise Training in Pregnancy for obese women (ETIP): study protocol for a randomised controlled trial. Trials. 2011;12:154 Epub 2011/06/21. PubMed Central PMCID: PMC3148988. 10.1186/1745-6215-12-154 21682869 PMC3148988

[28] Exercise during pregnancy and the postpartum period. Clin Obstet Gynecol. 2003;46(2):496–9. Epub 2003/06/17. 12808399 10.1097/00003081-200306000-00028

[29] ACOG Committee opinion. Number 267, January 2002: exercise during pregnancy and the postpartum period. Obstet Gynecol. 2002;99(1):171–3. Epub 2002/01/05. 11777528 10.1016/s0029-7844(01)01749-5

[30] Borg G. [Physical training. 3. Perceived exertion in physical work]. Lakartidningen. 1970;67(40):4548–57. 5477775

[31] Rasmussen KM, Abrams B, Bodnar LM, Butte NF, Catalano PM, Maria Siega-Riz A. Recommendations for weight gain during pregnancy in the context of the obesity epidemic. Obstetrics and gynecology. 2010;116(5):1191–5. PubMed Central PMCID: PMC4288953. 10.1097/AOG.0b013e3181f60da7 20966705 PMC4288953

[32] Mosteller RD. Simplified calculation of body-surface area. N Engl J Med. 1987;317(17):1098 10.1056/NEJM198710223171717 3657876

[33] Christiansen T, Paulsen SK, Bruun JM, Pedersen SB, Richelsen B. Exercise training versus diet-induced weight-loss on metabolic risk factors and inflammatory markers in obese subjects: a 12-week randomized intervention study. Am J Physiol Endocrinol Metab. 2010;298(4):E824–31. Epub 2010/01/21. 10.1152/ajpendo.00574.2009 20086201

[34] Wolff S, Legarth J, Vangsgaard K, Toubro S, Astrup A. A randomized trial of the effects of dietary counseling on gestational weight gain and glucose metabolism in obese pregnant women. International journal of obesity (2005). 2008;32(3):495–501. Epub 2008/01/30. 18227847 10.1038/sj.ijo.0803710

[35] Seneviratne SN, Parry GK, McCowan LM, Ekeroma A, Jiang Y, Gusso S, et al Antenatal exercise in overweight and obese women and its effects on offspring and maternal health: design and rationale of the IMPROVE (Improving Maternal and Progeny Obesity Via Exercise) randomised controlled trial. BMC Pregnancy Childbirth. 2014;14:148 Epub 2014/04/29. PubMed Central PMCID: PMCPMC4002538. 10.1186/1471-2393-14-148 24767604 PMC4002538

[36] Hopkins SA, Baldi JC, Cutfield WS, McCowan L, Hofman PL. Exercise training in pregnancy reduces offspring size without changes in maternal insulin sensitivity. J Clin Endocrinol Metab. 2010;95(5):2080–8. 10.1210/jc.2009-2255 20335449

[37] Barakat R, Perales M, Bacchi M, Coteron J, Refoyo I. A program of exercise throughout pregnancy. Is it safe to mother and newborn? Am J Health Promot. 2014;29(1):2–8. Epub 2013/11/10. 10.4278/ajhp.130131-QUAN-56 24200335

[38] Tanvig M, Vinter CA, Jorgensen JS, Wehberg S, Ovesen PG, Beck-Nielsen H, et al Effects of lifestyle intervention in pregnancy and anthropometrics at birth on offspring metabolic profile at 2.8 years: results from the Lifestyle in Pregnancy and Offspring (LiPO) study. J Clin Endocrinol Metab. 2015;100(1):175–83. 10.1210/jc.2014-2675 25343235

[39] Barakat R, Pelaez M, Cordero Y, Perales M, Lopez C, Coteron J, et al Exercise during pregnancy protects against hypertension and macrosomia: randomized clinical trial. Am J Obstet Gynecol. 2016;214(5):649 e1–8. 26704894 10.1016/j.ajog.2015.11.039

[40] Thangaratinam S, Rogozinska E, Jolly K, Glinkowski S, Duda W, Borowiack E, et al Interventions to reduce or prevent obesity in pregnant women: a systematic review. Health Technol Assess. 2012;16(31):iii–iv, 1–191. PubMed Central PMCID: PMC4781281. 10.3310/hta16310 22814301 PMC4781281

[41] Lau C, Rogers JM, Desai M, Ross MG. Fetal programming of adult disease: implications for prenatal care. Obstet Gynecol. 2011;117(4):978–85. 10.1097/AOG.0b013e318212140e 21422872

[42] Barakat R, Pelaez M, Montejo R, Luaces M, Zakynthinaki M. Exercise during pregnancy improves maternal health perception: a randomized controlled trial. Am J Obstet Gynecol. 2011;204(5):402 e1–7. 21354547 10.1016/j.ajog.2011.01.043

[43] Nascimento SL, Surita FG, Parpinelli MA, Siani S, Pinto e Silva JL. The effect of an antenatal physical exercise programme on maternal/perinatal outcomes and quality of life in overweight and obese pregnant women: a randomised clinical trial. BJOG. 2011;118(12):1455–63. Epub 2011/09/08. 10.1111/j.1471-0528.2011.03084.x 21895947

[44] Hopkins SA, Baldi JC, Cutfield WS, McCowan L, Hofman PL. Effects of exercise training on maternal hormonal changes in pregnancy. Clin Endocrinol (Oxf). 2011;74(4):495–500. Epub 2011/01/05. 21198740 10.1111/j.1365-2265.2010.03964.x

[45] Barakat R, Ruiz JR, Stirling JR, Zakynthinaki M, Lucia A. Type of delivery is not affected by light resistance and toning exercise training during pregnancy: a randomized controlled trial. Am J Obstet Gynecol. 2009;201(6):590.e1–6. Epub 2009/07/18. 19608151 10.1016/j.ajog.2009.06.004

[46] Thompson JM, Irgens LM, Skjaerven R, Rasmussen S. Placenta weight percentile curves for singleton deliveries. BJOG. 2007;114(6):715–20. Epub 2007/05/23. 10.1111/j.1471-0528.2007.01327.x 17516963

[47] Hayward CE, Lean S, Sibley CP, Jones RL, Wareing M, Greenwood SL, et al Placental Adaptation: What Can We Learn from Birthweight:Placental Weight Ratio? Front Physiol. 2016;7:28 Epub 2016/02/24. PubMed Central PMCID: PMCPMC4742558. 10.3389/fphys.2016.00028 26903878 PMC4742558

[48] Risnes KR, Romundstad PR, Nilsen TI, Eskild A, Vatten LJ. Placental weight relative to birth weight and long-term cardiovascular mortality: findings from a cohort of 31,307 men and women. Am J Epidemiol. 2009;170(5):622–31. Epub 2009/07/30. 10.1093/aje/kwp182 19638481

[49] Hemachandra AH, Howards PP, Furth SL, Klebanoff MA. Birth weight, postnatal growth, and risk for high blood pressure at 7 years of age: results from the Collaborative Perinatal Project. Pediatrics. 2007;119(6):e1264–70. Epub 2007/06/05. 10.1542/peds.2005-2486 17545358

[50] Barker DJ, Bull AR, Osmond C, Simmonds SJ. Fetal and placental size and risk of hypertension in adult life. BMJ. 1990;301(6746):259–62. Epub 1990/08/04. PubMed Central PMCID: PMCPMC1663477. 2390618 10.1136/bmj.301.6746.259 PMC1663477

[51] Barakat R, Stirling JR, Lucia A. Does exercise training during pregnancy affect gestational age? A randomised controlled trial. Br J Sports Med. 2008;42(8):674–8. Epub 2008/06/17. 10.1136/bjsm.2008.047837 18552370

[52] The Apgar Score. Pediatrics. 2015;136(4):819–22. Epub 2015/09/30. 10.1542/peds.2015-2651 26416932

[53] Ehrenstein V. Association of Apgar scores with death and neurologic disability. Clin Epidemiol. 2009;1:45–53. PubMed Central PMCID: PMCPMC2943160. 20865086 10.2147/clep.s4782 PMC2943160

[54] Choi J, Fukuoka Y, Lee JH. The effects of physical activity and physical activity plus diet interventions on body weight in overweight or obese women who are pregnant or in postpartum: a systematic review and meta-analysis of randomized controlled trials. Prev Med. 2013;56(6):351–64. PubMed Central PMCID: PMC3670949. 10.1016/j.ypmed.2013.02.021 23480971 PMC3670949

[55] Finlayson G, Bryant E, Blundell JE, King NA. Acute compensatory eating following exercise is associated with implicit hedonic wanting for food. Physiol Behav. 2009;97(1):62–7. 10.1016/j.physbeh.2009.02.002 19419671

[56] Stafne SN, Salvesen KA, Romundstad PR, Eggebo TM, Carlsen SM, Morkved S. Regular exercise during pregnancy to prevent gestational diabetes: a randomized controlled trial. Obstetrics and gynecology. 2012;119(1):29–36. Epub 2011/12/21. 10.1097/AOG.0b013e3182393f86 22183208

[57] Lutsiv O, Mah J, Beyene J, McDonald SD. The effects of morbid obesity on maternal and neonatal health outcomes: a systematic review and meta-analyses. Obes Rev. 2015;16(7):531–46. 10.1111/obr.12283 25912896

[58] Khashan AS, Kenny LC. The effects of maternal body mass index on pregnancy outcome. Eur J Epidemiol. 2009;24(11):697–705. Epub 2009/08/05. 10.1007/s10654-009-9375-2 19653107

[59] Athukorala C, Rumbold AR, Willson KJ, Crowther CA. The risk of adverse pregnancy outcomes in women who are overweight or obese. BMC pregnancy and childbirth. 2010;10:56 PubMed Central PMCID: PMC2949787. 10.1186/1471-2393-10-56 20849609 PMC2949787

[60] Wolfe LA, Hall P, Webb KA, Goodman L, Monga M, McGrath MJ. Prescription of aerobic exercise during pregnancy. Sports Med. 1989;8(5):273–301. 2692121 10.2165/00007256-198908050-00003

[61] Di Mascio D, Magro-Malosso ER, Saccone G, Marhefka GD, Berghella V. Exercise during pregnancy in normal-weight women and risk of preterm birth: a systematic review and meta-analysis of randomized controlled trials. American journal of obstetrics and gynecology. 2016;215(5):561–71. Epub 2016/10/30. 10.1016/j.ajog.2016.06.014 27319364

