# Supplementary material for: Effect of supervised exercise training during pregnancy on neonatal and maternal outcomes among overweight and obese women. Secondary analyses of the ETIP trial: A randomised controlled trial
Source: PLoS One. 2017 Mar 21;12(3):e0173937. doi: 10.1371/journal.pone.0173937 (PMC5360254; doi:10.1371/journal.pone.0173937)
Supplement: S1 Table — Neonatal outcomes at delivery for the per-protocol exercise group and the control group. Continuous data is presented as mean and standard deviation (SD) with comparison between groups as mean difference with 95% confidence interval (CI) and p-value. Dichotomous data is presented as number (n) and percent (%) and comparison between groups as odds ratio (OR), with 95% confidence interval (CI) and p-value. (DOCX) [file pone.0173937.s001.docx]

| **Neonatal Outcomes** | **Exercise group**  **n = 19** | **Control group**  **n = 36** | **Between-group differences** | | |
| --- | --- | --- | --- | --- | --- |
|  | *Mean SD/*  *n (%)* | *Mean SD/*  *n (%)* | *Mean diff/*  *OR* | *95 % CI* | *p-value* |
| Birth weight (g) | 3742 ± 652 | 3912 ± 413 | -170.04 | -458.42, 118.43 | 0.24 |
| Birth weight > 4000 g | 7 (37) | 19 (53) | 1.9 | 0.61, 5.98 | 0.40 |
| Gestational age (weeks) | 39.1 ± 1.8 | 39.5 ± 1.3 | -0.48 | -1.32, 0.37 | 0.27 |
| Length (cm) | 50.7 ± 1.8 | 51.1 ± 1.9 | -0.41 | -1.51, 0.70 | 0.47 |
| Head circumference (cm) | 35.8 ± 1.7 | 35.8 ± 1.5 | 0.02 | -0.89, 0.93 | 0.97 |
| Abdominal circumference (cm) | 31.9 ± 2.3 | 31.9 ± 2.1 | 0.08 | -1.37, 1.22 | 0.91 |
| Upper arm circumference (cm) | 10.9 ± 0.9 | 11.4 ± 1.0 | -0.52 | -1.09, 0.05 | 0.07 |
| BMI at birth (kg/m^2^) | 14.8 ± 1.5 | 15.0 ± 1.3 | -0.13 | -0.94, 0.67 | 0.74 |
| BSA (m^2^) | 0.23 ± 0.02 | 0.24 ± 0.02 | -0.003 | -0.011, 0.004 | 0.38 |
| Skinfold thickness triceps | 6.1 ± 2.1 | 6.3 ± 2.1 | -0.18 | -1.46, 1.10 | 0.78 |
| Skinfold thickness subscapularis | 5.4 ± 1.6 | 5.7 ± 1.9 | -0.24 | -1.30, 0.82 | 0.65 |
| Apgar score 1 minute | 8.2 ± 1.3 | 8.3 ± 1.7 | -0.12 | -0.99, 0.78 | 0.43 |
| Apgar score 5 minute | 9.5 ± 0.6 | 9.4 ± 1.2 | 0.70 | -0.47, 0.70 | 0.58 |
| Placenta weight (g) | 678.1 ± 175.9 | 666.7 ± 128.6 | 11.43 | -77.00, 99.83 | 0.80 |
| Placental weight ratio | 0.18 ± 0.02 | 0.17 ± 0.03 | 0.01 | -0.005, 0.02 | 0.19 |
| Transfer to NICU | 1 (0) | 3 (9) | 1.8 | 0.17, 18.64 | 1.00 |
| Preterm birth | 1 (5) | 0 (0) | - | - | 0.33 |
| *Missing:* Differs between the variables and varies between 1 and 4 in the exercise group and between 4 and 11 in the control group.  *Statistics:*  Continuous variables were analysed by Independent Samples t-test, dichotomous variables by Fisher’s Exact Test and Pearson Chi-Square. Apgar-score were analysed by Nonparametric Tests, Mann-Whitney U.  Abbreviations: BMI: Body mass index. BSA: Body Surface Area. NICU: Neonatal Intensive Care Unit.  *Definitions:* Gestational age: Weeks between the first day of the mother’s last menstrual period and the day of delivery. Placental weight ratio: Placenta weight divided on birth weight. Preterm birth: Delivery before gestational week 37. | | | | | |

**Supplementary Table 1**. Neonatal outcomes at delivery for the per-protocol exercise group and the control group. Continuous data is presented as mean and standard deviation (SD) with comparison between groups as mean difference with 95% confidence interval (CI) and p-value. Dichotomous data is presented as number (n) and percent (%) and comparison between groups as odds ratio (OR), with 95% confidence interval (CI) and p-value.
