# Supplementary material for: Effect of supervised exercise training during pregnancy on neonatal and maternal outcomes among overweight and obese women. Secondary analyses of the ETIP trial: A randomised controlled trial
Source: PLoS One. 2017 Mar 21;12(3):e0173937. doi: 10.1371/journal.pone.0173937 (PMC5360254; doi:10.1371/journal.pone.0173937)
Supplement: S2 Table — Maternal outcomes at delivery for the per-protocol exercise group and the control group. Continuous data is presented as mean and standard deviation (SD), with comparison between groups are as mean difference with 95% confidence interval (CI) and p-value. Dichotomous data is presented as number (n) and percent (%), with comparison between groups as odds ratio (OR), with 95% confidence interval (CI) and p-value. (DOCX) [file pone.0173937.s002.docx]

**Supplementary table 2**. Maternal outcomes at delivery for the per-protocol exercise group and the control group. Continuous data is presented as mean and standard deviation (SD), with comparison between groups are as mean difference with 95% confidence interval (CI) and p-value. Dichotomous data is presented as number (*n*) and percent (%), with comparison between groups as odds ratio (OR), with 95% confidence interval (CI) and p-value.

| **Maternal Outcomes** | **Per-protocol exercise group**  **n = 19** | **Control group**  **n = 36** | **Between-group differences** | | |
| --- | --- | --- | --- | --- | --- |
|  | *Mean SD/*  *n (%)* | *Mean SD/*  *n (%)* | *Mean diff/*  *OR* | *95 % CI* | *p-value* |
| Length of hospital stay (days) | 5.1± 1.7 | 4.5 ± 1.5 | 0.55 | -0.36, 1.46 | 0.45 |
| *Mode of delivery:* |  |  |  |  |  |
| Normal vaginal delivery | 11 (58) | 24 (69) | 1.59 | 0.50, 5.01 | 0.55 |
| Operative vaginal delivery | 5 (26) | 5 (14) | 0.47 | 0.12, 1.88 | 0.30 |
| Caesarean section | 4 (21) | 6 (17) | 0.78 | 0.19, 3.18 | 0.73 |
| Perineal tears, grade 3-4 | 2 (20) | 2 (10) | 0.42 | -0.05, 3.53 | 0.58 |
| Numbers less than n=19 in the exercise group and n=36 in the control groups were due to missing values. For mean length of hospital stay there were 4 missing in the exercise group and 5 in the control group.  *Statistics:*  Continuous variables were analysed by Independent Samples t-test, dichotomous variables by Fisher’s Exact Test and Pearson Chi-Square. | | | | | |
