# Supplementary material for: Effect of supervised exercise training during pregnancy on neonatal and maternal outcomes among overweight and obese women. Secondary analyses of the ETIP trial: A randomised controlled trial
Source: PLoS One. 2017 Mar 21;12(3):e0173937. doi: 10.1371/journal.pone.0173937 (PMC5360254; doi:10.1371/journal.pone.0173937)
Supplement: S3 Table — Baseline characteristics of all women included in the ETIP study. (DOCX) [file pone.0173937.s003.docx]

Table 1. Baseline characteristics of all women included in the ETIP study.

| **Baseline Characteristic** | **Exercise Group (*n =* 46)** | **Control Group (*n =* 45)** |
| --- | --- | --- |
| **Age (years)** | 31.3 ± 3.8 | 31.4 ± 4.7 |
| **Weight (kg)** | 95.3 ± 12.8 | 98.3 ± 14.2 |
| **Height (cm)** | 167.6 ± 5.9 | 167.1 ± 6.5 |
| **BMI (kg/m^2^)** | 33.9 ± 3.8 | 35.1 ± 4.6 |
| **Weight classification** |  |  |
| Overweight, BMI 28.0–29.9 kg/m^2^ | 3 (6.6%) | 5 (11.1%) |
| Class 1 obesity, BMI 30.0–34.9 kg/m^2^ | 28 (62.2%) | 19 (42.2%) |
| Class 2 obesity, BMI 35.0–39.9 kg/m^2^ | 11 (24.4%) | 15 (33.3%) |
| Class 3 obesity, BMI ≥ 40.0 kg/m^2^ | 3 (6.6%) | 6 (13.3%) |
| **Parity** |  |  |
| 0 | 22 (47.8%) | 19 (42.2%) |
| 1 | 19 (41.3%) | 19 (42.2%) |
| 2 | 5 (10.9%) | 4 (8.9%) |
| ≥3 | 0 (0.0%) | 3 (6.7%) |
| **Current smoking** | 2 (4.7%) | 4 (8.9%) |
| **Education** |  |  |
| Primary/secondary school | 1 (2.3%) | 3 (7.0%) |
| High school | 15 (34.1%) | 12 (27.9%) |
| University ≤4 y | 14 (31.8%) | 11 (25.6%) |
| University >4 y | 14 (31.8%) | 17 (39.5%) |
| **Currently employed** | 38 (82.6%) | 33 (73.3%) |
| **GDM** |  |  |
| WHO 2009 definition* | 4 (8.7%) | 4 (8.9%) |
| WHO/IADPSG 2013 definition** | 8 (18.2%) | 13 (29.5%) |
| **Maternal hypertension** | 3 (7.0%) | 4 (9.5%) |
| **Body composition***** |  |  |
| Fat mass (kg) | 40.0 ± 7.7 | 44.1 ± 10.3 |
| Fat mass (percent) | 43.1 ± 3.8 | 44.8 ± 5.5 |
| Fat-free mass (kg) | 52.4 ± 5.6 | 53.3 ± 6.1 |
| Fat-free mass (percent) | 56.9 ± 3.8 | 55.2 ± 5.5 |
| **Skinfold thickness** |  |  |
| Biceps area (mm) | 20.3 ± 8.9 | 22.3 ± 8.8 |
| Triceps area (mm) | 28.8 ± 7.0 | 31.2 ± 7.2 |
| Subscapular area (mm) | 30.5 ± 8.6 | 33.1 ± 7.9 |
| **Blood pressure** |  |  |
| Systolic blood pressure (mm Hg) | 126.3 ± 20.9 | 127.9 ± 12.9 |
| Diastolic blood pressure (mm Hg) | 75.0 ± 10.0 | 78.0 ± 8.4 |
| **Blood measurements** |  |  |
| Fasting glucose (mmol/l) | 4.6 ± 0.4 | 5.0 ± 0.8 |
| 120-min glucose (mmol/l) | 6.2 ± 1.1 | 6.1 ± 1.6 |
| Insulin (pmol/L) | 158.3 ± 62.5 | 150.0 ± 70.8 |
| HbA1c (percent) | 5.2 ± 0.3 | 5.4 ± 0.4 |
| Insulin C-peptide (nmol/l) | 0.7 ± 0.7 | 0.7 ± 0.4 |
| Triglycerides (mmol/l) | 1.4 ± 0.4 | 1.5 ± 0.2 |
| Ferritin (pmol/L) | 147.4 ± 97.5 | 84.9 ± 49.4 |
| HDL cholesterol (mmol/l) | 1.6 ± 0.3 | 1.8 ± 0.3 |
| LDL cholesterol (mmol/l) | 3.0 ± 0.9 | 3.0 ± 0.5 |
| Total cholesterol (mmol/l) | 5.0 ± 1.3 | 5.1 ± 1.2 |
| Hemoglobin (g/L) | 127.0 ± 100.0 | 126 ± 8.0 |
| High-sensitivity CRP (mg/l) | 8.4 ± 3.5 | 10.2 ± 5.0 |
| HOMA2-IR | 2.6 ± 1.2 | 2.5 ± 1.2 |

Observed data are presented as mean ± standard deviation or number of participants (percent). Missing: number of participants with missing data in each group is 0 to 3 for all variables except for body composition, where 14 are missing in the exercise group and 12 in the control group.

*Fasting plasma glucose ≥ 7.0 mmol/l or 120-min plasma glucose ≥ 7.8 mmol/l.

**Fasting plasma glucose ≥ 5.1 mmol/l or 120-min plasma glucose ≥ 8.5 mmol/l.

***Body composition was measured by air displacement plethysmography (BOD POD).
